# Fourth-Generation Epac-Based FRET Sensors for cAMP Feature Exceptional Brightness, Photostability and Dynamic Range: Characterization of Dedicated Sensors for FLIM, for Ratiometry and with High Affinity

**DOI:** 10.1371/journal.pone.0122513

**Published:** 2015-04-14

**Authors:** Jeffrey Klarenbeek, Joachim Goedhart, Aernoud van Batenburg, Daniella Groenewald, Kees Jalink

**Affiliations:** 1 Division of Cell Biology, The Netherlands Cancer Institute, Amsterdam, The Netherlands; 2 Section of Molecular Cytology, Swammerdam Institute for Life Sciences, University of Amsterdam, The Netherlands; 3 van Leeuwenhoek Centre of Advanced Microscopy, Amsterdam, The Netherlands; The Beatson Institute for Cancer Research, UNITED KINGDOM

## Abstract

Epac-based FRET sensors have been widely used for the detection of cAMP concentrations in living cells. Originally developed by us as well as others, we have since then reported several important optimizations that make these sensors favourite among many cell biologists. We here report cloning and characterization of our fourth generation of cAMP sensors, which feature outstanding photostability, dynamic range and signal-to-noise ratio. The design is based on mTurquoise2, currently the brightest and most bleaching-resistant donor, and a new acceptor cassette that consists of a tandem of two cp^173^Venus fluorophores. We also report variants with a single point mutation, Q270E, in the Epac moiety, which decreases the dissociation constant of cAMP from 9.5 to 4 μM, and thus increases the affinity ~ 2.5-fold. Finally, we also prepared and characterized dedicated variants with non-emitting (dark) acceptors for single-wavelength FLIM acquisition that display an exceptional near-doubling of fluorescence lifetime upon saturation of cAMP levels. We believe this generation of cAMP outperforms all other sensors and therefore recommend these sensors for all future studies.

## Introduction

In the past decade fluorescent biosensors have become important tools to study signalling events in living cells. FRET (Fluorescence or Förster Resonance Energy Transfer) is the non-radiative energy transfer from a donor to a suitable acceptor fluorophore and occurs only when donor and acceptor fluorophores are in close proximity (<10 nm). FRET is commonly read out as either Sensitized Emission (SE) of the acceptor, or as a decrease in fluorescent lifetime of the donor (Fluorescent Lifetime Imaging or FLIM). SE can be recorded using relative simple and widely available equipment; however it is not quantative unless endpoint calibrations are done or rather involved corrections are carried out. FLIM, on the other hand, is a quantitative and precise way to measure FRET, because donor lifetime decreases reflect increased FRET efficiency directly. However, since lifetimes of fluorophores are in the order of nanoseconds, it requires dedicated and complex equipment.

In 2004 the first Epac (Exchange protein directly activated by cAMP)-based FRET sensors for the detection of the ubiquitous second messenger cyclic AdenosineMonoPhosphate (cAMP) were developed[1–3]. In essence, these sensors consist of (part of) the cAMP-binding Rap-1 activating protein Epac, sandwiched between suitable donor- and acceptor fluorescent proteins. Whereas some colleagues opted to use only the cAMP binding domain of Epac1, we rather used the full length Epac1[3] because it displayed a larger conformational change, and thereby FRET change. To prevent the construct from affecting downstream effector proteins, we deleted the membrane-targeting DEP-sequence (ΔDEP mutant) and made a catalytically dead version (CD)[4,5]. Over the last decade, we and others described further optimization of these FRET sensors.

Much effort was aimed at further increasing the FRET span and S/N of the sensors by systematic exchange of fluorophores on both donor and acceptor side of the construct[4–6]. In van der Krogt et al, 2008, we reported extensively on the various considerations that contribute to optimizing FRET-sensor performance and detection. In that paper, we compared a wide range of constructs with different acceptors, donors and linkers. We analysed performance of these constructs with respect to S/N, dynamic range, brightness and affinity, but also less commonly addressed properties such as photobleaching, photochromism, speckle formation and suitability for FLIM- and ratio-detection. The main findings those studies may be summarized as follows.

1. Different readout methods really favour different design features of FRET sensors. FLIM detection benefits from high donor QY but does not profit from high acceptor QY. In contrast, for ratiometrical methods acceptor QY should be high, whereas the effect of donor QY on fluorescence ratios is really somewhat complex. On one hand, high donor QY extends the Förster radius and increases FRET, thereby affecting the dynamic range and S/N of the sensor, but on the other hand it also leads to increased donor leakthrough into the acceptor channel. The latter counteracts the cAMP-induced changes in signal in the acceptor channel and thus deteriorates the ratio changes.
2. FRET decreases rapidly with increasing distance, but the donor-acceptor orientation (κ^2^, a factor that ranges between 0 and 4) plays an equally important role. κ ^2^ is typically unknown but often assumed to be 2/3 for freely rotating fluorophores) For that reason, we tested the full range of circular permutated acceptors in our constructs [7,8]. In these extensive comparisons, as well as in follow-up studies by us and others we found that with very few exceptions ^cp173^YFP performs best, irrespective of the donor or FRET construct used.
3. Many FRET sensors, including Epac sensors, occasionally give rise to unwanted highly fluorescent "speckles" that appear throughout the cytosol after transfection. Speckle formation typically starts a few days after transfection, it depends on expression levels and is cell type-dependent. It has been proposed that speckle formation is due to formation of insoluble aggregates which may subsequently be cleared by autophagy [9]. We reasoned that aggregates may arise because of the presence of two fluorescent proteins in the FRET sensors, which increases the affidity between constructs. Indeed, speckle formation was largely diminished when the single acceptor, YFP or Venus, was replaced with a tandem yellow acceptor. We presume [5] that this is due to dimerization *within* the double acceptor moiety, which effectively circumvents the avidity increase, although direct evidence for this notion is lacking.
4. We also reported on red-shifted FRET variants as well as on variants that were better suited for FLIM detection[4,5]. Based on these and other criteria, we identified a panel of different Epac sensors, each pitched at different applications, that have been used in over 100 studies to date.

In this brief report we present cloning and characterization of the next generation of cAMP biosensors. These biosensors incorporate the new donor mTurquoise2[10], and also an optimized FRET acceptor, namely a tandem of the circular permutated version ^cp173^Venus. We also present a sensor with a novel, improved non-emitting (dark) acceptor [11,12] that is optimized for FLIM detection. This sensor should also be optimal for the simultaneous detection of multiple different FRET sensors in the same cell. Finally, we introduced a point mutation in Epac that increases the affinity for cAMP[13,14] by 2 to 3-fold. Since these 'fourth generation' Epac sensors significantly outperform earlier constructs in all aspects tested we suggest that they be used for all future studies.

## Materials and Methods

### Cell culture and transfection

Hek293T embryonal kidney cells (American Type Culture Collection crl-1573) N1E-115 mouse neuroblastoma cells (crl-2263) and U2OS human osteosarcoma cells (ATCC HTB-96), a kind gift of Martijn Luijsterburg (LUMC, Leiden, The Netherlands), were cultured in DMEM supplemented with 10% FCS and antibiotics at 37°C in a humidified incubator with 5% CO_2_. Cells were seeded on 24-mm coverslips in six-well plates and transfected with 1 μg DNA per well using PEI (HEK293T and N1E-115) (Polysciences Warrington, USA) or lipofectamine (U2OS) (LifeTechnologies Bleiswijk, The Netherlands).

### Constructs and materials

Using standard molecular cloning and PCR techniques, starting with mTurquoiseΔ-Epac(CD, ΔDEP)-^cp173^Venus-Venus and mTurquoise2 we generated mTurquoise2Δ-Epac(CD, ΔDEP)-^cp173^Venus-Venus, Epac-S^H126^, by inserting a stripped version of mTurquoise2, mTurquoise2Δ, lacking GITLGMDELYK, with forward PCR primer GATCGGCGGCCGCAATGGTGAGCAAGGGCGAGGAG and reverse primer AAAGGATATCGGGCGGCGGTCACGAACT. PCR products and plasmids were cut with NotI (Bioke Leiden, The Netherlands) and Eco32I (Fisher Scientific Landsmeer, The Netherlands). The Q270E mutation was introduced by cutting the Epac-S^H126^ with PshAI en BstEII (both Bioke Leiden, The Netherlands) inserting annealed oligo’s forward primer GTGACCCATGGCAAGGGGCTGGTGACCACCCTGCATGAGGGAGATGATTTTGGAGAGCTGGCTCTGGTCAATGATGCACCCCGGGCAGCCACCATCATCCTGCGAGAAGACAA and reverse primer TTGTCTTCTCGCAGGATGATGGTGGCTGCCCGGGGTGC-ATCATTGACCAGAGCCAGCTCTCCAAAATCATCTCCCTCATGCAGGGTGGTCACCAGCCCCTTGCCATGG. Underlined are the mutated nucleotides. The dark variants of Venus were made by mutating tyrosine-145 to tryptophan[11,12] with forward primer CACAA-GCTGGAGTACAACTGGAACAGCCACAACGTC and reverse primer GACGTTGTGG-CTGTTCCAGTTGTACTCCAGCTTGTG. All constructs were checked by sequence analysis. Double acceptor fluorophores were made as described previously[5].

IBMX and forskolin were obtained from (Calbiochem-Novabiochem Corp. La Jolla, USA). PGE1 and isoproterenol were obtained from Sigma-Aldrich (Zwijndrecht, The Netherlands).

### Confocal microscopy

**Fig 1:**
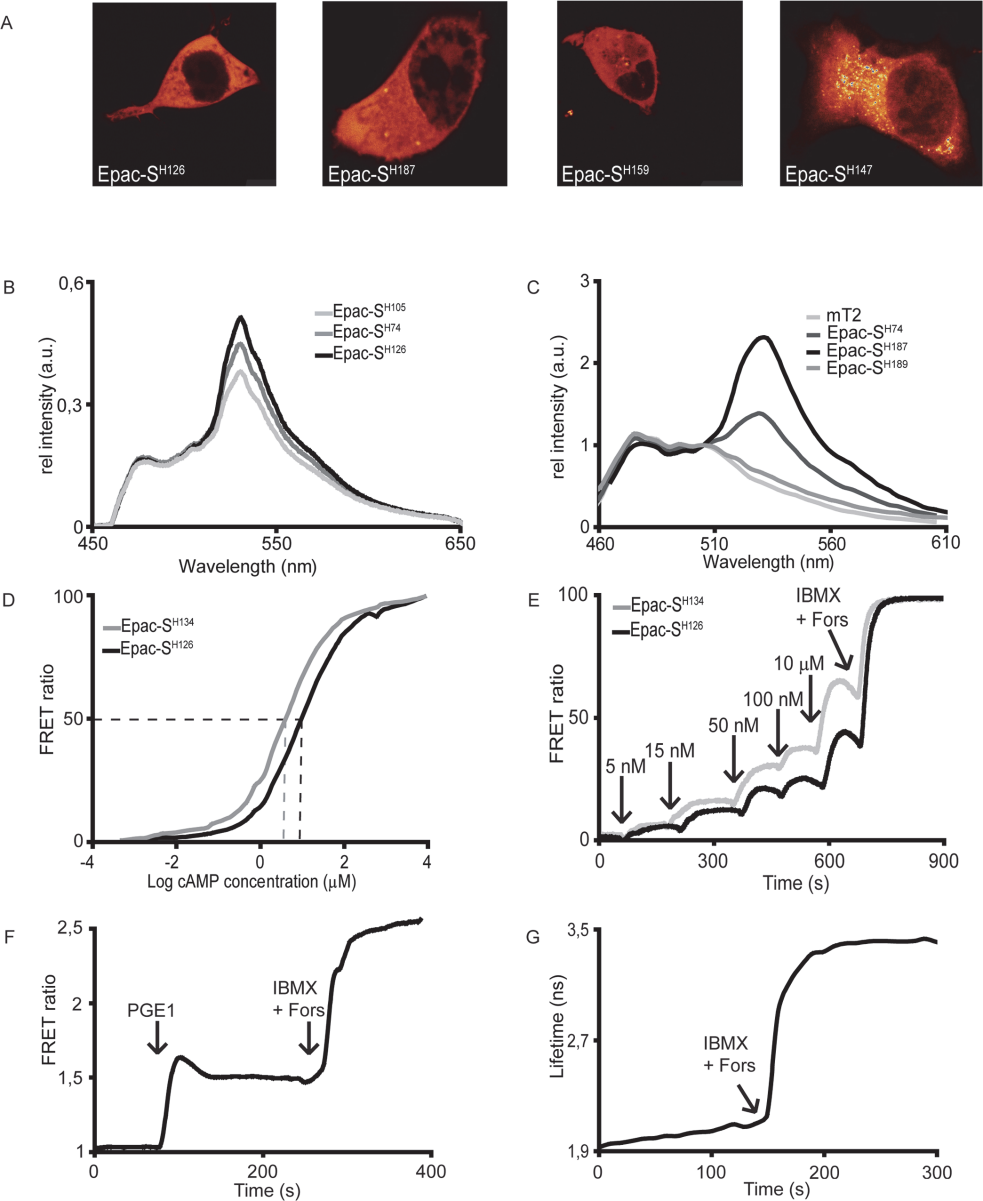
Characterization of novel FRET sensors. A. Localization of the FRET-sensors: N1E-115 cells transfected with mTurquoise2Δ-Epac(CD, ΔDEP)-^cp173^Venus-Venus (Epac-S^H126^); mTurquoise2Δ-Epac(CD, ΔDEP, Q270E)-td^cp173^Venus (Epac-S^H187^); mTurquoise2Δ-Epac(CD, ΔDEP)-td^cp173^Dark Venus (Epac-S^H159^) and mTurquoise2Δ-Epac(CD, ΔDEP)-^cp174^Cit (Epac-S^H147^). Images were taken 18 hours after transfection. B. FRET efficiency of constructs with different donors. Emission spectra of U2OS cells expressing constructs with donors Cerulaen3 (Epac-S^H105^); mTurquoise (Epac-S^H74^) or mTurquoise2 (Epac-S^H126^) were acquired while exciting at 436 nm. Spectra are normalized to CFP intensity after correction for expression levels (using Venus brightness, excited at 500 nm as detailed in M&M). C. Spectra of constructs with various acceptors. Shown are spectra from N1E-115 cells expressing Epac with acceptor ^cp173^Venus-Venus (Epac-S^H134^); td^cp173^Venus (Epac-S^H187^) or td^cp173^Dark Venus (Epac-S^H189^), illuminated with a 442 nm laser. Emission spectra were normalized to isosbestic point at 505 nm. D. Calibration curves for normal and high-affinity sensors. Shown are the average of three independent calibrations performed on cell lysates of HEK293T cells expressing the indicated constructs. cAMP was titrated in under continuous stirring. The sensors were excited at 420 +/- 3 nm and emission was measured at 530 +/- 10 nm and 490 +/- 10 nm for YFP and CFP respectively. Ratios were calculated as YFP over CFP and were normalized between baseline 0% and maximum response 100%. E. In-vivo experiment revealing the difference between high- and normal affinity sensors (Epac-S^H126^ or Epac-S^H134^) expressed in Hek293T cells. Isoproterenol induces a graded increase in cAMP levels in these cells and was added in increasing amounts as indicated. Signals were normalized between baseline 0% and maximum response 100%. Representative experiment out of 3 repeats. F. Typical FRET time-lapse trace in N1E-115 cells expressing Epac-S^H187^. After recording a baseline, at t = 90 s PGE1 (5 μM) was added, and at t = 250 s IBMX (100 μM) and Forskolin (25 μM) were added for calibration. G. A FLIM-FRET time-lapse recording from N1E-115 cells expressing Epac-S^H189^. A baseline was followed by addition of IBMX (100 μM) and Forskolin (25 μM) after 140 seconds.

Experiments were performed in HEPES buffered saline (containing in mM 140 NaCl, 5 KCl, 1 MgCl_2_, 1 CaCl_2_, 10 glucose, 10 HEPES) pH = 7.2 at 37°C. Images were taken using a Leica TCS-SP5 confocal point-scanning microscope (Mannheim; Germany) using a 63x, 1.4 N.A. oil immersion objective. Donor excitation was with the 442 nm HeCd laser. The spectral emission scans presented in Fig 1C were taken in xyλ mode, scanning between 465 nm and 605 nm with a 5-nm step size.

### Spectral imaging microscopy

The spectral imaging approach for determining brightness of the FRET sensors was performed as described before[15]. Briefly, U2OS cells transfected with EPAC sensors were excited with light from a mercury Arc lamp passed through a 436/10 nm excitation filter. The emission was long-pass filtered (LP460) and detected with an imaging spectrograph (Imspector V7, Specim, Finland) and CCD camera (ORCA ER, Hamamatsu, Japan). Subsequently, an image was acquired from the same field of view to quantify relative YFP intensity (excitation at 500/20 nm and emission passed through a 534/20 nm filter). Each full emission spectrum was divided by the YFP emission, thereby correcting for differences in expression levels. The average normalized emission spectrum of at least twelve cells was calculated for each construct.

### Ratiometric FRET analysis in vivo

Cells grown on coverslips were placed on a temperature-controlled (37° C) inverted Nikon Diaphot microscope and excited at 425 nm. Donor and acceptor emission was detected simultaneously with two photomultipliers, using a 505 nm beamsplitter and optical filters: 470 ± 20 nm (CFP) and 530 ± 25 nm (YFP). Signals were digitized and FRET was expressed as the ratio between donor and acceptor signals. The FRET ratio was set at 1 at the onset of the experiment. Cells were stimulated with 25 μM forskolin and 100 μM IBMX to maximally raise the cAMP levels. Data from a minimum of 15 cells over three experiments are presented as mean ± s.e.m.

### Ratiometric FRET analysis in vitro

Hek293T cells were transfected with Epac-S^H126^ or Epac-S^H134^ washed with PBS and resuspended in a hypotonic buffer (PBS: H2O 1: 2) and homogenized with a Downs piston. The homogenate was centrifuged for 10 minutes at 4° C. Isotonicity was restored using a concentrated stock of PBS. The homogenate was diluted 10 times in buffer containing (in mM) 150 KCl, 5 NaCl, 1 MgCl_2_ 10 HEPES pH 7.2 in the stirred cuvet of a PTI Quantamaster dual channel spectrofluorimeter (Lawrenceville, NJ). Excitation was at 420 +/- 3 nm. Slits were adjusted so that emission intensities of YFP (530 +/- 10 nm) and CFP (490 +/- 10 nm) were equal (initial ratio = 1). FRET was expressed as deviations from this initial ratio.

### Fluorescence Lifetime Imaging Microscopy

Frequency-domain FLIM measurements were performed with LI-FLIM software and hardware, attached to a Leica DMIRE2 wide-field microscope. A 1 Watt 442 nm LED, modulated at 40 MHz, and a 63x objective (Plan Apochromat NA 1.3 glycerin) were used. The emitted light (480 +/- 15 nm) was collected from transiently transfected cells. Lifetimes were calculated from 12 phase images, with an exposure time of 50–400 ms seconds. These experiments were carried out at 37°C. Initial (pre-stimulation) lifetimes showed considerable variability due to variations in basal cAMP levels. For characterization of FRET span, cells with low initial cAMP levels were selected and lifetime was compared prior to and after stimulation with IBMX and Forskolin. Differences in lifetime are expressed as percentage increase relative to the unstimulated cells.

## Results and Discussion

### Experimental design

In recent studies, we tested performance of a large variety of donor-acceptor combinations in cAMP FRET sensors[4,5]. We showed that a circular permuted version of Venus, ^cp173^Venus, is favoured over Venus as well as other permuted versions of Venus as acceptor in Epac1-based cAMP sensors. We also found that inclusion of a tandem of ^cp173^Venus/Venus as acceptor had the additional advantage that it strongly diminished formation of bright aggregates of sensors (speckles) seen in some cells. The hitherto best construct from these previous studies, construct H74 (mTurquoiseΔ-Epac(CD, ΔDEP)-^cp173^Venus-Venus) has been extensively used by the community and was used as a reference against which to judge performance of the new constructs.

For the current study, all constructs were transfected 18–48 hours prior to performing experiments. We used human embryonal kidney Hek293T cells, mouse neuroblastoma N1E-115 cells and human osteosarcoma U2OS cells for these studies. Expression was seen predominantly in the cytosol although as described before [4,5,16] sometimes some enrichment at the nuclear envelop is observed, especially when the sensors were expressed at low levels (Fig 1A). For all new constructs, we checked that neither the changes in fluorophores nor mutations in the Epac backbone affected the distribution of the constructs in the cell.

Constructs were compared with respect to cAMP-induced change in donor/acceptor ratio, change in lifetime as detected by frequency-domain FLIM and other photophysical characteristics, including maturation, bleaching resistance and sensitivity to pH changes. Constructs that performed poorly in either of these parameters are omitted from this report.

### Nomenclature

**Fig 2:**
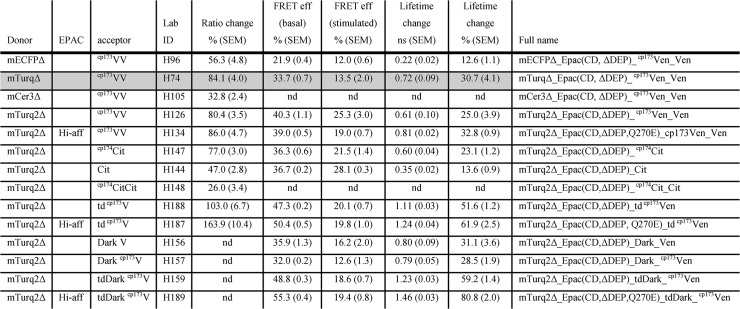
Summary of FRET changes of the constructs mentioned in this study. Mentioned are construct details (column 1–3), unique Lab ID (column 4), % ratio change (expressed as cAMP-induced change from an initial baseline ratio of 1, column 5) with its Standard Error of Mean, FRET efficiency E in rest (column 6) with SEM, E after saturation of the sensor with IBMX and Forskolin with SEM (column 7), absolute change of lifetime in ns with SEM (column 8) and % change in lifetime with SEM (column 9). Column 10 contains the fully descriptive name. As mentioned in the main text, we will from now on for brevity name the sensors "Epac-S^LabID^", for example, Epac-S^H96^ for mECFPΔ_Epac(CD, ΔDEP)_cp^173^Ven_Ven. In gray is the reference construct Epac-S^H74^. Abbreviations: nd, not determined; mCer3, monomeric Cerulean 3; mECFP, monomeric enhanced CFP; mTurq, monomeric Turquoise fluorescent protein; Hi-aff, High-affinity (Q270E mutant); ^cp173^V, circular permutation of the fluorescent protein Venus(etc); td ^cp173^V (etc), tandem of two ^cp173^V fluorophores; ^cp173^VV, tandem of cpVenus and Venus; CD, catalytically dead mutant (T781A & F782A amino acids of the Epac1 wild type protein); ΔDEP, deletion of the DEP domain to prevent membrane localization. Note that unlike Venus, Citrine contained the A206K monomerizing mutation. FRET efficiency E was calculated as E = 1-τ_DonorAcceptor_/τ_Donor_ using τ_Donor_ = 4.10 ns for mTurquoise2 and 3.70 ns for mTurquoise1.

As we have published and given out many different Epac-based cAMP sensor constructs, the need arises for a consistent naming convention. In a previous manuscript, we used e.g. ^T^Epac^VV^ as shorthand for a construct encompassing mTurquoiseΔ-Epac(CD, ΔDEP)-^cp173^Venus-Venus. With the introduction of other varieties, including high-affinity versions and dark acceptors, such shorthand notations are no longer practical. We therefore will stick to the following nomenclature. All Epac sensors coming from our lab will be named Epac-S followed by the unique identifier assigned to the construct in our lab, e.g., Epac-S^H187^. If the specific properties of a particular sensor are relevant for a manuscript, we propose to use the more elaborate abbreviated name that describes its features in some details (see Fig 2). In manuscripts where two or more sensors need to be used side-by-side, we suggest using the base name followed by a single superscript mnemonic that clarifies its specific use, e.g. Epac-S^N^ versus Epac-S^Hi^ for normal- and high-affinity versions, or Epac-S^Ratio^ versus Epac-S^FLIM^ for ratiometry-optimized versus FLIM-optimized sensors, respectively. Mentioning the unique identifier name in the materials and methods section should suffice to prevent confusion.

### Donors

Two new CFP variants that were recently reported to have significantly increased brightness and photostability were tested: mTurquoise2, which introduced the I147F mutation, and Cerulean3 which contains the same mutation but differs in a number of other residues[10,17]. The enhanced brightness allows using dimmer excitation and therefore significantly diminishes phototoxic effects, while the higher photostability makes it possible to image for longer times. To examine the brightness of Epac sensors with these donors, we used a spectral imaging approach as was described before [15] (see Materials and Methods). Transfected U2OS cells were excited at 436 nm and emission spectra were measured. Subsequently, the Venus emission intensity was acquired at 520 nm excitation and the individual spectra were normalised to this intensity to correct for differences in protein level. This allows direct comparison of the brightness and sensitized emission of single chain FRET biosensors. As can be inferred from Fig 1B, the relative brightness and sensitized emission of mTurquoise2Δ-Epac(CD, ΔDEP)-^cp173^Venus-Venus (hereafter called Epac-S^H126^) is higher than our previously reported best sensor, mTurquoiseΔ-Epac(CD, ΔDEP)-^cp173^Venus-Venus (Epac-S^H74^) as could be expected from the extremely high quantum yield of mTurquoise2. Furthermore, this donor also has a much higher FRET efficiency than Cerulean3 in the construct mCerulean3Δ-Epac(CD, ΔDEP)-^cp173^Venus-Venus (Epac-S^H105^) as can be concluded from the much higher emission of the acceptor (Fig 1B). The finding that mTurquoise2 performs better in a FRET sensor agrees with data showing that in cells, mTurquoise2 is brighter than mCerulean3[10]. Therefore, we conclude that mTurquoise2 is the donor of choice for Epac sensors.

### Acceptors

In a recent study, Salonikidis et al reported that the YFP variant Citrine [18] appears quite effective in diminishing speckle formation[6]. We therefore tested both Citrine (Epac-S^H144^) and ^cp174^Citrine (Epac-S^H147^) as acceptors in constructs that have mTurquoise2 as donor. In contrast to the previous report[6], three days after transfection both constructs showed speckle formation in all three cells types tested (Fig 1A right panel and data not shown). Therefore we introduced a tandem acceptor consisting of ^cp174^Citrine and Citrine in the Epac sensor (Epac-S^H148^). In line with our previous observations, this tandem acceptor construct strongly decreased speckle formation, but it showed only moderate FRET changes as measured by ratiometry (Fig 2). Although in some cases Citrine appears to give good results[19], our data do not support superiority of Citrine as acceptor in these sensors, as none of the Citrine constructs outperformed our reference Epac-S^H74^ (see Fig 2).

We also included a new tandem Venus acceptor in our study in mTurquoise2Δ-Epac(CD, ΔDEP)-^cp173^Venus-^cp173^Venus (Epac-S^H188^). This construct displayed much higher initial FRET efficiency by spectral characterization (Fig 1C) as well as by FLIM detection (Fig 2). Both in time-lapse ratiometry and FLIM experiments (Fig 2), Epac-S^H188^ proved superior, with FRET ratio changes following addition of forskolin and IBMX around 100%. FLIM recording confirmed the superior FRET change of Epac-S^H188^.

### Dark acceptors

Dark, i.e., non-emitting, acceptors are useful for FLIM sensor construction. First, dark acceptors allow selection of a larger part of the donor spectrum for FLIM measurements, thereby increasing brightness and diminishing phototoxicity. Second, FRET constructs with dark acceptors open the possibility of simultaneous recording from different FLIM sensors in the same cell as they occupy a smaller part of the spectrum. Dark versions of both Venus and ^cp173^Venus were prepared by introducing the point mutation Y145W as described for YFP. These were then used to prepare Epac sensors with a Dark Venus acceptor (Epac-S^H156^), a Dark ^cp173^Venus acceptor (Epac-S^H157^) and a tandem Dark ^cp173^Venus acceptors (Epac-S^H159^). Low acceptor emission of the dark acceptor constructs was checked by spectroscopy (Fig 1C shows Epac-S^H189^ as representative example for all Dark Venus variants). Interestingly, these constructs showed very high basal (i.e., in the unstimulated state) FRET efficiency of up to 55% (Fig 2). In addition, all constructs showed very robust FRET changes after stimulation with IBMX and Forskolin, with the tandem Dark ^cp173^Venus construct Epac-S^H159^ outperforming all other constructs in FLIM recordings (Fig 2, column 8 and 9). We therefore recommend Epac-S^H159^ for future FLIM experiments.

### High-affinity sensors

A glutamine to glutamic acid mutation at position 270 was described to increase the affinity of full-length Epac1 for cAMP by 2.5 fold[13,14]. Introducing this mutation (at corresponding position 357) in Epac-S^H126^, we generated the high-affinity version Epac-S^H134^. Affinity of both sensors for cAMP was checked in vitro in cell homogenates (Fig 1D). The K_d_ of Epac-S^H126^, 9.5 +/- 0.3 μM was in reasonable agreement with values reported earlier (14 +/- 2 μM). The K_d_ of the high-affinity version Epac-S^H134^ was 4.0 +/- 0.1 μM. This value is somewhat higher than that was published before, but it still presents an approximately 2.4-fold increase as compared to the maternal construct. Note that Dao et al[13] used GST-purified full length Epac1 instead of cell homogenates. We failed at isolating our sensors using either His- or GST-tags because constructs rapidly lost FRET after or during purification (data not shown). In living cells, too, we could show higher affinity of Epac-S^H134^, as shown in Fig 1E. Increasing amounts of the β-adrenergic receptor agonist isoproterenol were added to gradually increase cAMP in Epac-S^H126^ or Epac-S^H134^ expressing Hek293T cells and signals were normalised to their minimal and maximal response for comparison. At all concentrations of isoproterenol, Epac-S^H134^ showed a higher normalized ratio change.

We next also prepared and characterized high-affinity versions of our best bright (Epac-S^H188^) and dark acceptor (Epac-S^H159^) sensors, Epac-S^H187^ and Epac-S^H189^, respectively. As documented in Fig 1F and Fig 2, Epac-S^H187^ significantly outperformed the parental construct Epac-S^H188^ in ratio experiments. Somewhat surprisingly, this indicates that the Q270E mutation in Epac may cause a conformational shift that further increases FRET in these constructs. A similar effect was seen in cells transfected with the dark acceptor high-affinity FRET construct, Epac-S^H189^ that displayed an astonishing increase in lifetime of 1.46 ns, a full 80% change (Fig 1G). In all other aspects, these constructs performed equal to the parental constructs.

## Concluding Remarks

Several new Epac based cAMP sensors were made and analyzed for performance. We tested brightness, photostability, fluorophore maturation, basal- and cAMP-induced FRET, dynamic range and signal-to-noise. We also compared non-spectroscopic properties including speckle formation, affinity, pH sensitivity and cellular localization. Only constructs that performed at least as good as the reference construct Epac-S^H74^ in all aspects tested were further characterized.

Our studies identified significantly improved FRET sensors dedicated for specific use. All new constructs feature mTurquoise2 as the donor. Tandem repeats of ^cp173^Venus proved optimal as acceptors. We may summarize our results as follows.

1. The best cAMP sensors for ratiometry and spectral detection are Epac-S^H188^ (normal affinity) and Epac-S^H187^ (high affinity). The increased affinity for cAMP is particularly useful for cells that display limited changes in cAMP, such as neurons.
2. In FLIM experiments, dark acceptors outperform their fluorescent counterparts. For lifetime determinations, we thus recommend Epac-S^H159^ (normal affinity) and Epac-S^H189^ (high-affinity). These sensors also free up part of the detection spectrum, in principle facilitating simultaneous readout of two or more FRET sensors in the same cell.
3. If only a single sensor is to be chosen (e.g., when transgenic animals are prepared), we recommend the bright-acceptor constructs H188 and H187 as these also perform quite well in FLIM experiments.

All constructs were tested in many (typically, >> 15) experiments on several different days. Nevertheless, small but systematic bias may occur in certain measurements for several reasons. We already mentioned that donor leakthrough may affect the ratio change negatively for high QY donors or for constructs with relatively low basal FRET efficiency. On the other hand, in FLIM measurements constructs with very high basal FRET efficiency tend to be rather dim (because FRET leads to reduced donor emission) and hence the effects of autofluorescence and (small) leakthrough of acceptor emission in the donor channel may be more prominent, offsetting the data. Thus, though there is good correlation between ratio changes and lifetime changes, there is no strict 1:1 correspondence between the two as has been noted before[5]. Furthermore, for lack of reliable tools to decrease cellular cAMP levels, our methods assume that resting levels of cAMP are too low to give detectable binding to the sensors.In line with that, we observed considerable day-to-day variability of lifetimes in unstimulated cells, in particular when high-affinity versions were used. This indicates that the reported FRET spans in Fig 2, if anything, may be underestimated for these constructs.

Note that for expression in e.g. primary neurones several of our previously published sensors were prepared in viral backbones, such as pSin-Rep (Epac-S^H151^) pAdeno (Epac-S^H170 & H171^ for normal and high affinity, respectively) and pLenti (Epac-S^H183^), for a summary, see Fig 2 and Table 1. For completeness, Table 1 also includes our red-shifted FRET sensor Epac-S^H81^ and Epac-S^H94^ with GFP as the donor. For sequence information, a complete list of available sensors and information on the newest unpublished additions to the family, please check http://research.nki.nl/jalinklab/.

**Table 1:** Recommended constructs for new experiments.

Donor	EPAC	acceptor	Lab ID	Application	Full name
mTurq2Δ		td ^cp173^V	H188	SE/FLIM	mTurq2Δ_Epac(CD,ΔDEP)_td ^cp173^Ven
mTurq2Δ	Hi-aff	td ^cp173^V	H187	SE/FLIM low cAMP levels	mTurq2Δ_Epac(CD,ΔDEP, Q270E)_td ^cp173^Ven
mTurq2Δ		dark td ^cp173^V	H159	FLIM	mTurq2Δ_Epac(CD,ΔDEP)_tdDark_ ^cp173^Ven
mTurq2Δ	Hi-aff	dark td ^cp173^V	H189	FLIM low cAMP levels	mTurq2Δ_Epac(CD,ΔDEP, Q270E)_tdDark_ ^cp173^Ven
GFPΔ		mRFP	H81	SE/FLIM red-shifted	GFPΔ_EPAC(CD,ΔDEP)_mRFP
GFP		mCherry	H94	SE/FLIM red-shifted	GFP_EPAC(CD,ΔDEP)_mCherry
mTurq2Δ		^cp173^VV	H170	SE/FLIM Adenoviral infection	pAdeno mTurq2Δ_EPAC(CD,ΔDEP)_ ^cp173^Ven_Ven
mTurq2Δ	Hi-aff	^cp173^VV	H171	SE/FLIM Adenoviral infection	pAdeno mTurq2Δ _EPAC(CD,ΔDEP, Q270E)_ ^cp173^Ven_Ven
mTurqΔ		^cp173^VV	H183	SE/FLIM Lentiviral infection	pLVX-mTurqΔ_EPAC(CD,ΔDEP)_ ^cp173^Ven_Ven

Mentioned are construct details (column 1–3), Unique Lab ID (column 4), optimal application (Sensitized Emission, SE, or Fluorescence Lifetime IMaging, FLIM; column 5) and the fully descriptive name (column 6). Abreviations: GFP, Green Fluorescent Protein; mRFP, monomeric Red Fluorescent Protein; pAdeno, plasmid with Adenoviral backbone; pLVX, plasmid with LentiViral eXpression backbone; for other abbreviations see Fig 2. The lentiviral construct, H183) was prepared by dr. J. Karczewski (Wageningen University, NL) and has not been separately evaluated on our equipment.

## Supporting Information

S1 Data Supporting information. (ZIP)
