# Supplementary figures and images for: Fourth-Generation Epac-Based FRET Sensors for cAMP Feature Exceptional Brightness, Photostability and Dynamic Range: Characterization of Dedicated Sensors for FLIM, for Ratiometry and with High Affinity
Source: PLoS One. 2015 Apr 14;10(4):e0122513. doi: 10.1371/journal.pone.0122513 (PMC4397040; doi:10.1371/journal.pone.0122513)

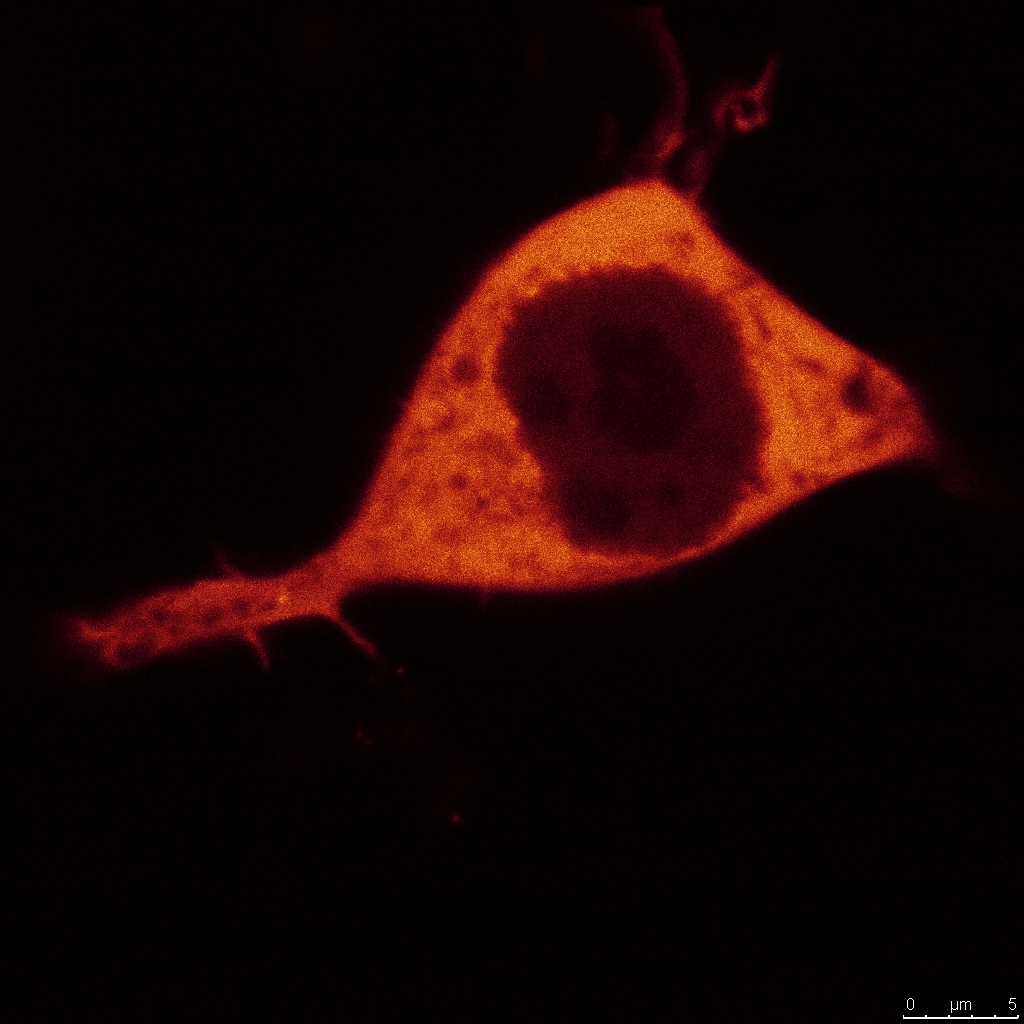

Supplement: S1 Data — (ZIP) [file pone.0122513.s001.zip › Raw/Confocal pictures/H126.jpg]

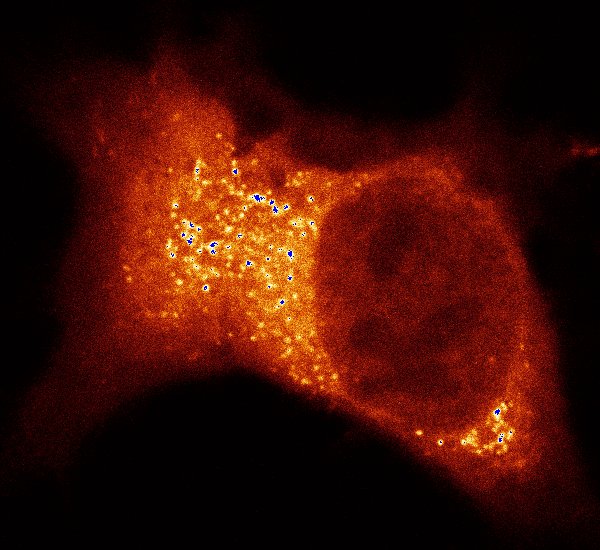

Supplement: S1 Data — (ZIP) [file pone.0122513.s001.zip › Raw/Confocal pictures/H147.jpg]

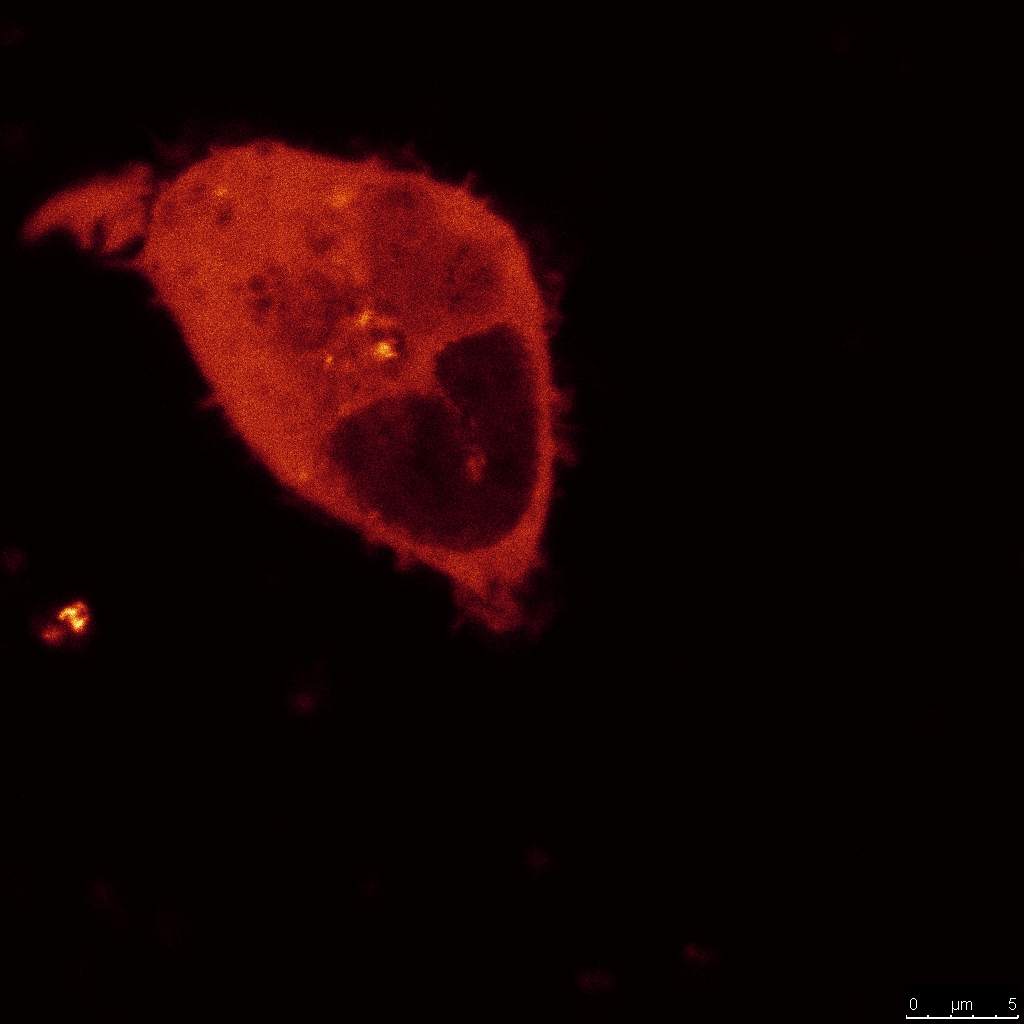

Supplement: S1 Data — (ZIP) [file pone.0122513.s001.zip › Raw/Confocal pictures/H159.jpg]

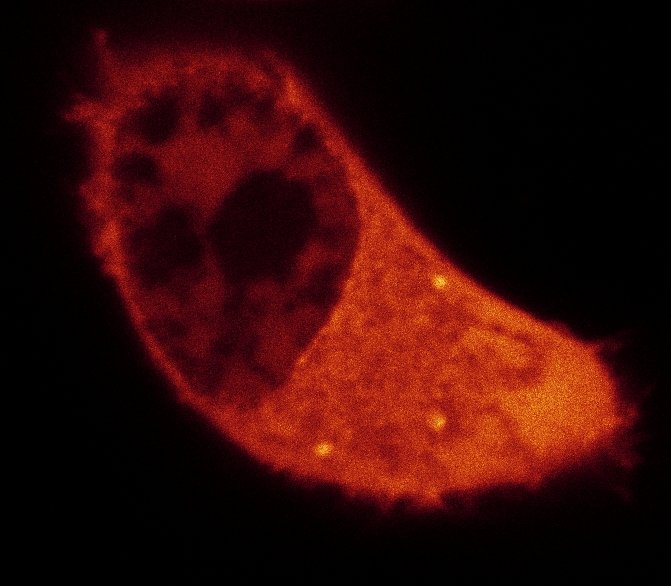

Supplement: S1 Data — (ZIP) [file pone.0122513.s001.zip › Raw/Confocal pictures/H187.jpg]
